# The Influence of Different Hamstrings Assemblies on the Final Graft Diameter in Anterior Cruciate Ligament Reconstruction

**DOI:** 10.1055/s-0044-1785518

**Published:** 2024-06-22

**Authors:** Lúcio Flávio Biondi Pinheiro, Marcos Henrique Frauendorf Cenni, Thiago César Ladeira Estefani, Allan José Lima Bueno, Thiago Penido Moreira Ferreira, Guilherme Cardoso Ferreira Pina

**Affiliations:** 1 Rede Mater Dei de Saúde, Belo Horizonte, MG, Brasil

**Keywords:** anterior cruciate ligament, bone-patellar tendon-bone grafts, hamstring tendons, knee

## Abstract

**Objective** This study aimed to compare gracilis and semitendinosus tendon graft diameters in anterior cruciate ligament (ACL) reconstruction using quadruple, quintuple, and sextuple assemblies. Another objective was to evaluate the percentage of patients in which each assembly type is possible, depending on the length of each free tendon.

**Methods** Seventy-one patients underwent ACL reconstruction using hamstring tendons. We measured the diameters of the quadruple, quintuple, and sextuple assemblies in all patients. We recorded tendon length and graft diameter from three assembly types.

**Results** Assembly comparison showed a statistically significant difference ( *p* < 0.001). In each assembly, graft diameter increased by 1 mm, a statistically significant value ( *p* < 0.001). In 2.8% of patients, the only potential assembly was the quadruple assembly because the free lengths of the 2 tendons removed were lower than 24 cm. The quintuple assembly was possible in 23.9% of subjects, as only the semitendinosus had a minimum length of 24 cm. The sextuple assembly was possible in 73.2% of patients because both tendons were at least 24 cm in length.

**Conclusion** A quintuple or sextuple assembly is possible in 97.2% of cases since the final graft length of at least 8 cm is statistically significant between comparisons.

## Introduction

Anterior cruciate ligament (ACL) reconstruction surgery is one of the most commonly performed procedures in orthopedics, with satisfactory clinical and functional outcomes. 1 2

Anterior cruciate ligament reconstruction uses different grafts according to the surgeon's choice. These grafts include autografts of the patellar tendon, quadriceps tendon, flexor tendons (hamstrings), peroneus longus, and several allogeneic grafts. 3

Medial flexor tendons have become popular due to their good clinical outcomes and lower knee morbidity compared with the patellar tendon. 4 However, ligament re-rupture with neoligament injury is a significant concern among surgeons. We know that graft thickness has a direct link to the re-rupture rate, and the flexor tendon diameter depends on the anatomy of each patient, potentially influencing the final quality of the graft assembly.

This study aimed to compare the diameter of hamstring grafts during ACL reconstruction using quadruple, quintuple, and sextuple assemblies by evaluating whether increasing the number of bands results in a thicker graft. Another objective was to evaluate the percentage of patients in whom each type of assembly is possible depending on the length of each free tendon.

## Materials and Methods

The Human Research Ethics Committee approved this study under the Opinion Certificate number CAAE 57891522.0.0000.5128.

Seventy-one patients (57 men and 14 women) underwent ACL reconstruction between March 2021 and November 2021 using the medial flexor, gracilis, and semitendinosus tendons as grafts. The patients' ages ranged from 15 to 54 years, and the mean age was 32.54 years. Graft preparation occurred on an auxiliary table during the surgery. The semitendinosus and gracilis tendons were harvested using a closed tendon harvester (striper) and removed from the muscle tissue residue. Tendon measurements occurred separately, at first, to obtain their initial length. Next, we prepared the quadruple, quintuple, and sextuple assemblies. For the quadruple graft, we folded the semitendinosus and gracilis tendons once (double); for the quintuple graft, we folded the semitendinosus twice (triple) and the gracilis once (double). In the sextuple assembly, the two tendons were double-folded (triple). We measured the diameters of the three assemblies in all patients, regardless of which one we would use in the surgery. The surgeon decided on the final assembly to use in the procedure, and this decision does not fall within the scope of this work.

The assembly chosen for surgery was the thickest graft with a minimum length of 8 cm. If the 2 tendons had a length greater than or equal to 24 cm, a sextuple assembly, with 2 triple tendons, was possible. When the semitendinosus tendon was greater than or equal to 24 cm in length and the gracilis tendon was less than 24 cm, we could use the quintuple assembly with a triple semitendinosus and a double gracilis graft. If the two tendons were less than 24 cm long, we used the quadruple assembly with the 2 double tendons.

## Results

The sample consisted of 71 patients and the same number of grafts. The quadruple assembly diameter is statistically smaller than the quintuple and sextuple assemblies ( *p* < 0.001), and comparisons between assembly pairs also showed a statistically significant difference ( *p* < 0.001). The average difference from the quadruple to the quintuple assembly and from the quintuple to the sextuple assembly was a statistically significant increase of 1 mm in the graft diameter ( *p* < 0.001) ( Table 1 ).

**Table 1:** Diameter (mm) comparison between assemblies.

Diameter (mm)	Mean	Standard deviation	Median	P25	P75	Minimum value	Maximum value	*P* -value*
Quadruple	8.0	0.8	8.0	7.5	8.5	6.0	10.0	
Quintuple	9.0	0.8	9.0	8.5	10.0	7.0	11.0	**< 0.001**
Sextuple	10.0	0.9	10.0	9.0	11.0	8.0	13.0	

*Kruskal-Wallis' test; Multiple comparisons: quadruple x quintuple; quadruple x sextuple; and quintuple x sextuple ( *p* < 0.001)

**Fig. 1:**
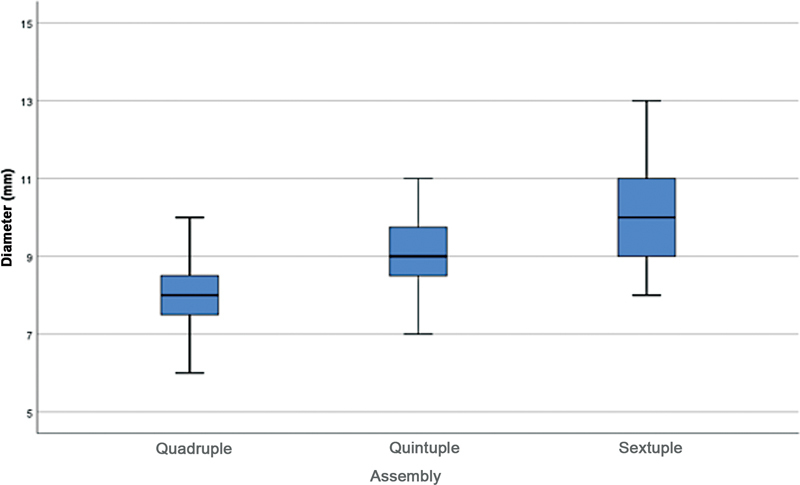
Diameter (mm) comparison between assemblies.

Fig. 1 illustrates this comparison.

Regarding the free length of the semitendinosus and gracilis tendons, we observed that only the quadruple assembly was viable in 2 patients (2.8%) as their length was lower than 24 cm. In 17 patients (23.9%), quadruple or quintuple assemblies was possible as the length of the semitendinosus graft was equal to or greater than 24 cm and the gracilis had less than 24 cm. Lastly, in 52 patients (73.2%), all assemblies were possible as the length of both tendons was equal to or greater than 24 cm ( Table 2 ).

**Table 2:** Potential assembly use per the free length of the semitendinosus and gracilis tendons

Assembly	n	%
Quadruple	2	2.8
Quintuple	17	23.9
Sextuple	52	73.2
Total	71	100.0

## Discussion

Reconstruction of the anterior cruciate ligament has produced good joint stability and functional outcomes. 3

The literature reports the relationship between graft diameter and its resistance and revision rate. 5 6 demonstrated a revision rate of 1.57% in patients with a graft diameter greater than 8 mm, 6.5% in patients with a graft between 7.5 and 8 mm, and 13.6% in patients with a graft smaller than 7 mm in diameter, reinforcing this relationship between re-rupture and graft thickness, consistent with the systematic review from Conte et al. 7

Our work showed that in most cases, that is, in 97% of patients, a quintuple or sextuple assembly with a graft diameter greater than 8 mm was possible.

Calvo et al. 8 reported an increase of 2 mm in graft diameter in a group of patients in whom they converted from quadruple to quintuple assembly due to insufficient thickness. This quadruple group had an average diameter of 7.2 mm, which increased to 9.2 mm after conversion into a quintuple assembly. These data are consistent with our study, in which there was also an increase in graft thickness from the quadruple to the quintuple assembly but of only 1 mm.

Fritsch et al. 9 described a technique modifying the graft preparation to increase its diameter, which is also in line with the results of our study. These authors report that more important than the absolute size of the graft is the ability to obtain a graft of the appropriate size for each subject based on their size and demand.

Attia et al. 10 evaluated 413 patients with grafts measuring at least 8 mm in diameter. The quadruple graft preparation had an average diameter of 8.25 mm, compared to 9.14 for the quintuple graft and 8.95 for the sextuple graft. The average difference in graft diameter was statistically significant when comparing the quadruple with the quintuple and quadruple with the sextuple assemblies, which is consistent with our study. However, there was no statistical difference in diameter when comparing the quintuple and sextuple assemblies. The failure rate in the quadruple assembly was higher than in the quintuple and sextuple assemblies, with 9.1% versus 2.3% and 2.7%, respectively. However, this difference did not reach statistical significance when the graft was at least 8 mm in diameter. 10

Tutkus et al. 2 evaluated 122 patients and used sextuple assemblies in 74 (60.7%) and quintuple assemblies in 48 (39.3%) of them. This finding is consistent with our study, in which we obtained quintuple assemblies in 23.9% and sextuple assemblies in 72.3% of patients. The average diameter of the quintuple assembly was 8.9/8.3 mm in men/women, and of the sextuple assembly was 9.3/8.5 mm in men/women, respectively. In 98.4% of cases, the quintuple or sextuple assembly was significantly thicker than 8 mm. This is also consistent with our study since quintuple or sextuple assemblies were possible in 97.2% of cases, with average diameters of 9 mm and 10 mm, respectively. 2

In a systematic review, Smith et al. 11 found an overall mean diameter of 8.4 mm for the quadruple graft and 9.1 mm for the quintuple graft, which is consistent with our study. However, there was no significant difference in failure rates or clinical outcomes between quadruple and quintuple grafts.

Due to the large variability in the diameter of hamstring grafts between patients, several authors have described the use of magnetic resonance imaging, weight, height, and gender to predict graft diameter before ACL reconstruction. 4 12 13

Unlike patellar and quadriceps tendon grafts, the diameter of the flexor tendons does not depend solely on the surgeon's ability to harvest the appropriate size but rather on the anatomical variations of each patient. According to Pinheiro Júnior et al., 13 height and gender are the most significant factors that help predict the diameter of the flexor tendons in their quadruple preparation. Our study did not consider these factors in predicting graft diameter. Likewise, we did not prospectively correlate graft thickness with the failure rate and clinical outcomes of surgery. These may be the subject of future studies.

## Conclusion

The diameter of the quadruple assembly measured was, on average, 1 mm smaller than the quintuple assembly, which was, on average, 1 mm smaller than the sextuple assembly ( *p* < 0.001). Assembly comparisons also showed a statistically significant difference ( *p* < 0.001). In 97.2% of cases, it was possible to use a quintuple or sextuple assembly with a minimum final graft length of 8 cm.
