# Supplementary material for: The Influence of Different Hamstrings Assemblies on the Final Graft Diameter in Anterior Cruciate Ligament Reconstruction
Source: Rev Bras Ortop (Sao Paulo). 2024 Jun 22;59(3):e393–6. doi: 10.1055/s-0044-1785518 (PMC11193592; doi:10.1055/s-0044-1785518)
Supplement: Related texts

# Research article: A influência de diferentes montagens dos tendões flexores no diâmetro final do enxerto na reconstrução do ligamento cruzado anterior

**Keywords:** enxertos osso-tendão patelar-osso, joelho, ligamento cruzado anterior, tendões isquiotibiais

## Abstract

**Objetivo** Buscamos comparar o diâmetro dos enxertos com utilização dos tendões grácil e semitendíneo na reconstrução do ligamento cruzado anterior (LCA) utilizando as montagens quádrupla, quíntupla e sêxtupla. Outro objetivo é avaliar em qual porcentagem de pacientes é possível cada tipo de montagem, em função do comprimento de cada tendão livre.

**Métodos** Setenta e um pacientes foram submetidos à reconstrução do LCA utilizando tendões isquitibiais. Foram medidos os diâmetros das montagens quádrupla, quíntupla e sêxtupla em todos pacientes. Registramos os comprimento dos tendões e o diâmetro do enxerto com os três tipos de montagens.

**Resultados** As comparações entre as montagens mostraram diferença estatisticamente significativa ( *p* < 0,001). A cada montagem, aumentou 1 mm o diâmetro do enxerto e isso foi estatisticamente significativo ( *p* < 0,001). Em 2,8% dos pacientes, somente a montagem quádrupla foi possível, pois os comprimentos livres dos 2 tendões retirados foram menores que 24 cm. Em 23,9% desses, foi possível a montagem quíntupla; pois somente o semitendíneo tinha comprimento mínimo de 24 cm e, em 73,2%, foi possível a montagem sêxtupla com o comprimento dos 2 tendões igual ou superior a 24 cm.

**Conclusão** Em 97,2% dos casos foi possível realizar a montagem quíntupla ou sêxtupla, já que o comprimento final do enxerto de no mínimo 8 cm apresenta diferença estatisticamente significante entre as comparações.

## Introdução

A cirurgia de reconstrução do ligamento cruzado anterior (LCA) é um dos procedimentos mais realizados na ortopedia, com resultados clínicos e funcionais satisfatórios. 1 2

Na técnica de reconstrução do LCA, utiliza-se diferentes enxertos, de acordo com a escolha do cirurgião; como o auto enxerto de tendão patelar, de tendão do quadríceps, de tendões flexores (isquiotibiais), fibular longo e diversas fontes de enxertos alógenos. 3

A utilização dos tendões flexores mediais tornou-se popular devido aos bons resultados clínicos e por possuir menor morbidade ao joelho em comparação com o tendão patelar. 4 No entanto, a re-ruptura ligamentar com lesão do neoligamento é uma preocupação importante entre os cirurgiões. Sabemos que a espessura do enxerto está diretamente ligada ao índice de re-ruptura, e o diâmetro dos tendões flexores depende da anatomia de cada paciente, o que pode influenciar na qualidade final da montagem do enxerto.

Este trabalho tem como objetivo comparar o diâmetro dos enxertos de isquiotibiais durante a reconstrução do ligamento cruzado anterior (LCA) utilizando montagens quádrupla, quíntupla e sêxtupla; avaliando se o aumento do número de bandas resulta em um enxerto de maior espessura. Outro objetivo é avaliar a porcentagem de pacientes em que é possível cada tipo de montagem, em função do comprimento de cada tendão livre.

## Materiais e métodos

Este estudo foi aprovado pelo Comitê de Ética em Pesquisa em Seres Humanos sob o número do Certificado de Liberação: (CAAE: 57891522.0.0000.5128).

Setenta e um pacientes (57 homens e 14 mulheres) foram submetidos à reconstrução do LCA entre março e novembro de 2021 utilizando como enxertos os tendões flexores mediais, grácil e semitendíneo. A idade dos pacientes variou de 15 a 54 anos com média de idade de 32,54 anos. O preparo dos enxertos foi realizado em mesa auxiliar durante a cirurgia. Os tendões semitendíneo e grácil foram colhidos usando um coletor de tendão fechado ( *striper* ) e retirados do resíduo de tecido muscular. Os tendões foram medidos em separado; inicialmente, para se obter o seu comprimento inicial. Depois foram feitas as montagens quádrupla, quíntupla e sêxtupla. No enxerto quádruplo, os tendões semitendíneo e grácil eram dobrados uma vez (duplos); no quíntuplo, o semitendíneo era dobrado duas vezes (triplo) e o grácil uma (duplo). Na montagem sêxtupla, os dois tendões eram dobrados duas vezes (triplos). Essas medidas de diâmetro foram coletadas nos três tipos de montagem em todos os pacientes, independente de qual delas seria utilizada na cirurgia. A decisão sobre a montagem final a ser utilizada na cirurgia ficou a cargo do cirurgião, não entrando no escopo deste trabalho.

Os parâmetros que influenciaram na decisão de qual das montagens utilizar na cirurgia foram baseados na obtenção do enxerto mais espesso que tivesse um comprimento mínimo de 8 centímetros. Se os dois tendões tinham comprimento maior ou igual a 24 cm, era possível a montagem sêxtupla, dois tendões triplos. Nos casos em que o tendão semitendíneo teve comprimento maior ou igual a 24 cm e o grácil menor que 24 cm, poderíamos utilizar a montagem quíntupla com semitendíneo triplo e grácil duplo. Nos casos em que os dois tendões possuíam comprimento inferior a 24 cm, utilizamos a montagem quádrupla com os dois tendões duplos.

## Resultados

A amostra é composta por 71 pacientes e mesmo número de enxertos. O diâmetro quádruplo é estatisticamente menor que o quíntuplo e o sêxtuplo, *p* < 0,001, e as comparações entre cada par de montagens também mostraram diferença estatisticamente significativa, *p* < 0,001. A diferença média da montagem quádrupla para quíntupla e da quíntupla para sêxtupla foi um aumento de 1 mm o diâmetro do enxerto e isso foi estatisticamente significativo *p* < 0,001 ( Tabela 1 ).

**Tabela 1:** Comparação dos diâmetros (mm) de acordo com as montagens

Diâmetro (mm)	Média	Desvio padrão	Mediana	P25	P75	Mínimo	Máximo	Valor de *p* *
Quádruplo	8,0	0,8	8,0	7,5	8,5	6,0	10,0	
Quíntuplo	9,0	0,8	9,0	8,5	10,0	7,0	11,0	**< 0,001**
Sêxtuplo	10,0	0,9	10,0	9,0	11,0	8,0	13,0	

*Teste Kruskal-Wallis; Comparações múltiplas: quádruplo X quíntuplo; quádruplo X sêxtuplo e quíntuplo X sêxtuplo ( *p* < 0,001)

A Fig. 1 abaixo mostra de forma ilustrativa essa comparação.

**Fig. 1:** Comparação dos diâmetros (mm) de acordo com as montagens.

Em relação ao comprimento livre dos tendões semitendíneo e grácil, foi observado que em 2 pacientes (2,8%) somente a montagem quádrupla seria viável, pois o comprimento dos 2 tendões era menor que 24 cm. Em 17 pacientes (23,9%), seria possível a montagem quádrupla ou quíntupla; pois o semitendíneo tinha comprimento igual ou superior a 24 cm e o grácil inferior a 24 cm. Por fim, em 52 pacientes (73,2%) todas as montagens seriam possíveis, pois o comprimento de ambos os tendões era igual ou superior a 24 cm ( Tabela 2 ).

**Tabela 2:** Possibilidades de montagem de acordo com o comprimento livre dos tendões semitendíneo e grácil

Montagem	n	%
Quádrupla	2	2,8
Quíntupla	17	23,9
Sêxtupla	52	73,2
Total	71	100,0

## Discussão

A reconstrução do LCA tem produzido boa estabilidade articular e bons resultados funcionais. 3

A literatura nos mostra a relação do diâmetro do enxerto com a sua resistência e a taxa de revisão. 5 6 Magnussen et al. demonstraram, em seus casos, um índice de revisão de 1,57% em pacientes com enxerto de diâmetro maior que 8 mm, 6,5% em pacientes com enxerto entre 7,5 e 8 mm, e 13,6% em pacientes com enxerto menor que 7 mm de diâmetro, reforçando essa relação da re-ruptura com a espessura do enxerto obtido, assim como Conte et al. em revisão sistemática. 7

Nosso trabalho mostrou que na grande maioria dos casos, em 97% dos pacientes, foi possível realizar uma montagem quíntupla ou sêxtupla com o diâmetro do enxerto superior a 8 mm.

Calvo et al. 8 relataram um aumento de 2 mm no diâmetro do enxerto em um grupo de pacientes em que converteram a montagem quádrupla para quíntupla devido a espessura insuficiente. Esse grupo quádruplo teve diâmetro médio de 7,2 mm e passou a ter 9,2 mm quando convertido na montagem quíntupla. Esses dados estão de acordo com o nosso estudo em que também houve um aumento da espessura do enxerto da montagem quádrupla para quíntupla; porém, somente de 1 mm.

Fritsch et al. 9 descrevem uma técnica em que modificaram o preparo do enxerto de forma a aumentar o seu diâmetro, o que também está de acordo com os resultados do nosso trabalho. Relataram que mais importante que o tamanho absoluto do enxerto é a habilidade de obter um enxerto de dimensão apropriada para cada indivíduo baseado em seu tamanho e demanda.

Attia et al. 10 avaliaram 413 pacientes com enxertos de no mínimo 8 mm de diâmetro. O preparo com enxerto quádruplo teve um diâmetro médio de 8,25 mm, comparado com 9,14 do grupo com enxerto quíntuplo e 8,95 do grupo com enxerto sêxtuplo. A diferença média no diâmetro dos enxertos foi estatisticamente significativa, quando comparados os grupos quádruplos com o quíntuplo e quádruplo com sêxtuplo, concordante com o resultado obtido no nosso trabalho. No entanto, não houve diferença estatística no diâmetro quando comparados os grupos quíntuplos com o sêxtuplo. O índice de falha no grupo quádruplo foi maior que nos grupos quíntuplo e sêxtuplo 9,1% x 2,3% e 2,7%, respectivamente. Todavia, essa diferença não alcançou uma significância estatística, quando o enxerto é reconstruído com no mínimo 8 mm de diâmetro. 10

Tutkus et al. 2 avaliaram 122 pacientes e obtiveram a montagem sêxtupla em 74 pacientes (60.7%) e a montagem quíntupla em 48 pacientes (39.3%). Esse achado é semelhante ao do nosso estudo, no qual obtivemos a montagem quíntupla em 23,9% e sêxtupla em 72,3% dos pacientes. O diâmetro da montagem quíntupla foi em média 8,9/8,3 mm em homens/mulheres, enquanto o da montagem sêxtupla foi 9,3/8,5 mm em homens/mulheres, respectivamente. Em 98,4% dos casos, a montagem quíntupla ou sêxtupla foi significativamente mais espessa que 8 mm. Isso também foi encontrado no nosso estudo; posto que, em que 97,2% dos casos, foi possível a montagem quíntupla ou sêxtupla com uma média de enxerto de 9 mm na quíntupla e 10 mm na sêxtupla. 2

Smith et al., 11 em revisão sistemática, encontraram uma média geral do diâmetro do enxerto quádruplo de 8,4 mm e do quíntuplo de 9,1 mm, resultado semelhante ao do nosso estudo. Entretanto, não houve diferença significativa nos índices de falhas nem nos resultados clínicos entre os enxertos quádruplos e quíntuplos.

Devido à grande variabilidade do diâmetro dos enxertos isquiotibiais entre os pacientes, vários autores descreveram o uso da ressonância magnética, peso, altura e sexo para fazer uma previsão do diâmetro do enxerto antes da reconstrução do LCA. 4 12 13

Ao contrário dos enxertos de tendões patelar e do quadríceps, o diâmetro dos tendões flexores não dependem somente da habilidade do cirurgião em coletar o tamanho adequado, mas sim das variações anatômicas de cada paciente. De acordo com Pinheiro Júnior et al., 13 a altura e o gênero são os fatores mais significativos que auxiliam a predizer o diâmetro dos tendões flexores em seu preparo quádruplo. No presente estudo, não levamos em consideração a avaliação desses fatores preditores do diâmetro do enxerto, nem uma avaliação prospectiva correlacionando a espessura do enxerto com o índice de falha e resultados clínicos da cirurgia. Esses podem ser objeto de estudos futuros.

## Conclusão

O diâmetro do enxerto quádruplo medido foi, em média, 1 mm menor que o quíntuplo, que foi em média, 1 mm menor que o sêxtuplo ( *p* < 0,001), e as comparações entre as montagens também mostraram diferença estatisticamente significativa ( *p* < 0,001). Em 97,2% dos casos foi possível realizar a montagem quíntupla ou sêxtupla, com o comprimento mínimo final do enxerto de 8 cm.
